# Image quality and related outcomes of the ShuntScope for catheter implantation in pediatric hydrocephalus—experience of 65 procedures

**DOI:** 10.1007/s00381-022-05776-1

**Published:** 2022-12-02

**Authors:** Anna Prajsnar-Borak, Fritz Teping, Joachim Oertel

**Affiliations:** Department of Neurosurgery, Saarland University Medical Center, Kirrbergerstraße, Building 90.5, D-66421 Homburg, Germany

**Keywords:** ShuntScope, Pediatric hydrocephalus, Endoscopy, Ventricular catheter, Shunting

## Abstract

**Purpose:**

Ventricular catheter implantation in pediatric hydrocephalus can become a highly challenging task due to abnormal anatomical configuration or the need for trans-aqueductal stent placement. Transluminal endoscopy with the ShuntScope has been invented to increase the rate of successful catheter placements. This study aims to evaluate ShuntScope’s image qualities and related surgical outcomes in the pediatric population.

**Methods:**

A retrospective analysis of all pediatric patients undergoing ventricular catheter placement using the ShuntScope from 01/2012 to 01/2022 in the author’s department was performed. Demographic, clinical, and radiological data were evaluated. The visualization quality of the intraoperative endoscopy was stratified into the categories of excellent, medium, and poor and compared to the postoperative catheter tip placement. Follow-up evaluation included the surgical revision rate due to proximal catheter occlusion.

**Results:**

A total of 65 ShuntScope-assisted surgeries have been performed on 51 children. The mean age was 5.1 years. The most common underlying pathology was a tumor- or cyst-related hydrocephalus in 51%. Achieved image quality was excellent in 41.5%, medium in 43%, and poor in 15.5%. Ideal catheter placement was achieved in 77%. There were no intraoperative complications and no technique-related morbidity associated with the ShuntScope. The revision rate due to proximal occlusion was 4.61% during a mean follow-up period of 39.7 years. No statistical correlation between image grade and accuracy of catheter position was observed (*p*-value was 0.290).

**Conclusion:**

The ShuntScope can be considered a valuable addition to standard surgical tools in treating pediatric hydrocephalus. Even suboptimal visualization contributes to high rates of correct catheter placement and, thereby, to a favorable clinical outcome.

**Supplementary Information:**

The online version contains supplementary material available at 10.1007/s00381-022-05776-1.

## Introduction

Ventricular catheter placement can be a highly challenging task in pediatric patients. Tumorous or cystic deformation and post-hemorrhagic or post-infectious conditions contribute to misleading anatomical configurations of the ventricular system. The correct ventricular catheter placement is a crucial condition for the shunt survival rate, even if statistical significance is not always achievable. When performing the standard freehand catheter insertion, intraoperative orientation on anatomical landmarks is limited. Subsequently, the rate of catheter misplacement is reported to be up to 35–45% in the literature [1–3]. The modern neurosurgeon can lean back on various technical innovations supporting intraoperative orientation and guidance. For ventricular catheter placement, neuronavigation, neuroendoscopy ultrasound, electromagnetic-based stereotaxy, or smartphone-based guiding tools have been used and reported as promising methods to improve surgical success rates [4–9]. However, these randomized studies have shown no significant benefit using the abovementioned supportive techniques. Despite the correct trajectory for implantation, the desire for high-quality visualization of intraventricular anatomy during surgery has also led to remarkable advances in endoscopic technology. Implementing the intraluminal fibreoptic ShuntScope has revolutionized catheter implantations in complex hydrocephalus cases. Several case series have reported favorable surgical outcomes by ensuring correct catheter placement with the ShuntScope [10–13]. However, data on intraluminal endoscopy is still limited to relatively small cohorts, often mixed with adults and children (Table 1). A detailed analysis of the quality of delivered images and the subsequent surgical success rate would be desirable. Therefore, this study aims to investigate related outcomes based on the quality of intraoperative endoscopic visualization in a large pediatric population.

**Table 1:** Literature review regarding endoscope-assisted VC placement

**Series**	**Number of procedures**	**Patients group**	**Endoscope used**	**Results**	**Follow-up**
Antes et al. [11]	6	Children and adults	ShuntScope	Aqueductal stenting performed in 4 out of 6 cases	NA
Antes et al. [10]	71	Children and adults	ShuntScope	Endoscopic application completed in 68 of 71 procedures, (failure rate, 5.8%)	31.6 months
Antes et al. [12]	12	Children and adults	ShuntScope	Optimal catheter placement achieved in 11 of 12 cases	NA
Senger et al. [13]	27	Children	ShuntScope	Precise catheter placement achieved in 26 of 27 patients In one case, the procedure was abandoned	2 months–4 years
Agraval et al. [14]	9	Children and adults	ShuntScope	Sufficient VC placement was achieved in all cases Intraventricular image quality was adequate in all cases	NA
Issa et al. [15]	29	Children and adults	ShuntScope	The success rate for optimal placement of VC was significantly higher than in the free hand technique (93.1% versus 67%, respectively)	19.56 months
Our series	65	Children	ShuntScope	Optimal catheter placement achieved in 77% of procedures Excellent image quality (41.5%), medium (43%) and poor (15.5%) ShuntScope failure rate 4.61%	Mean follow-up 39.7 months (1–114 months)
Kestle et al. [7]	Multicenter randomized trial 393 children; endoscopy in 194 patients	Children	Intra-catheter endoscope, not precisely defined	Shunt failure at 1 year was 42% in the endoscopic insertion group and 34% in the non-endoscopic group The time to first shunt failure was not different between the two groups	12 months
Roth et al. [16]	16	Children	NeuroPEN	14 procedures were technically successful Correct VC placement in 13 procedures	0 to 24 months
Jiaping et al. [17]	18	Children/adults	Rigid endoscope 10, flexible endoscope 8	Success rate in 82.7%	12 months

*NA* not available

## Methods

### General

Data of all patients who underwent CSF restoration surgery using the ShuntScope (Karl Storz GmbH & Co.KG, Tuttlingen, Germany) was acquired from the department’s internal database. Inclusion criteria for further analysis were the age < 18 years at the time of surgery and a complete dataset including medical documentation and follow-up, radiological studies, and intraoperative video documentation.

### Surgical technique and equipment

**Fig. 1:**
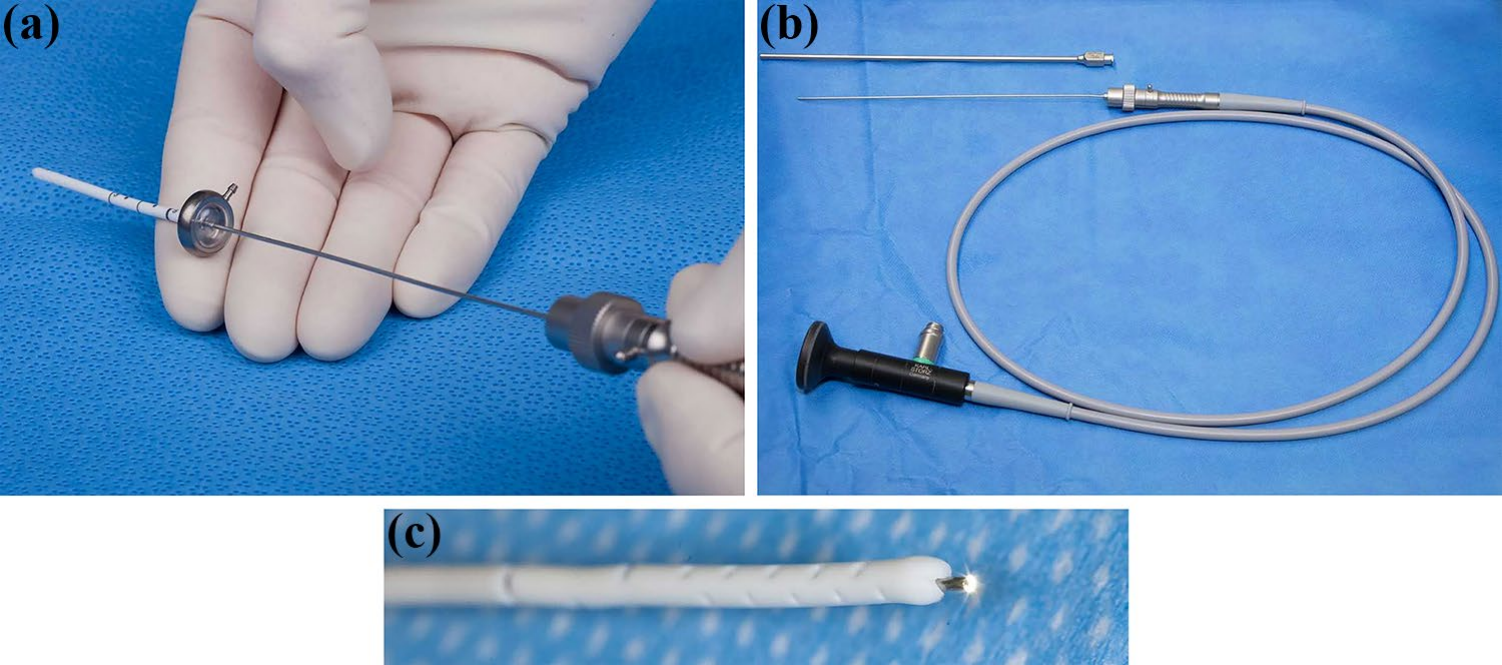
A semi-rigid ShuntScope, Karl Storz GmbH & Co.KG, Tuttlingen, Germany, with a length of 160 mm, an outer diameter of 1 mm, and an image resolution of 10,000 pixels

Indication for the usage of the ShuntScope was set on an individual base depending on the patient’s anatomical configuration and expected difficulty level. A semi-rigid ShuntScope Karl Storz GmbH & Co.KG, Tuttlingen, Germany, with a length of 160 mm, an outer diameter of 1 mm, and an image resolution of 10,000 pixels was applied (Fig. 1). To prepare the transluminal visualization, the catheter tip was incised. The ShuntScope was inserted into the ventricular catheter through a connected burr hole reservoir. Implantation of the ventricular catheter was conducted freehanded manually using the inserted ShuntScope as an intraluminal guidewire. After positioning, the ShuntScope was forwarded through the distal catheter tip incision, and intraventricular endoscopic inspection was performed. The final catheter positioning was adapted under endoscopic guidance. In the case of burr hole reservoir placement, the reservoir membrane was punctured with the ShuntScope. After the final positioning of the catheter, the ShuntScope was withdrawn. We did not observe a CSF leakage through the point of the punctured membrane of the reservoir.

A detailed description has been published before (Table 1).

### Data analysis

**Fig. 2:**
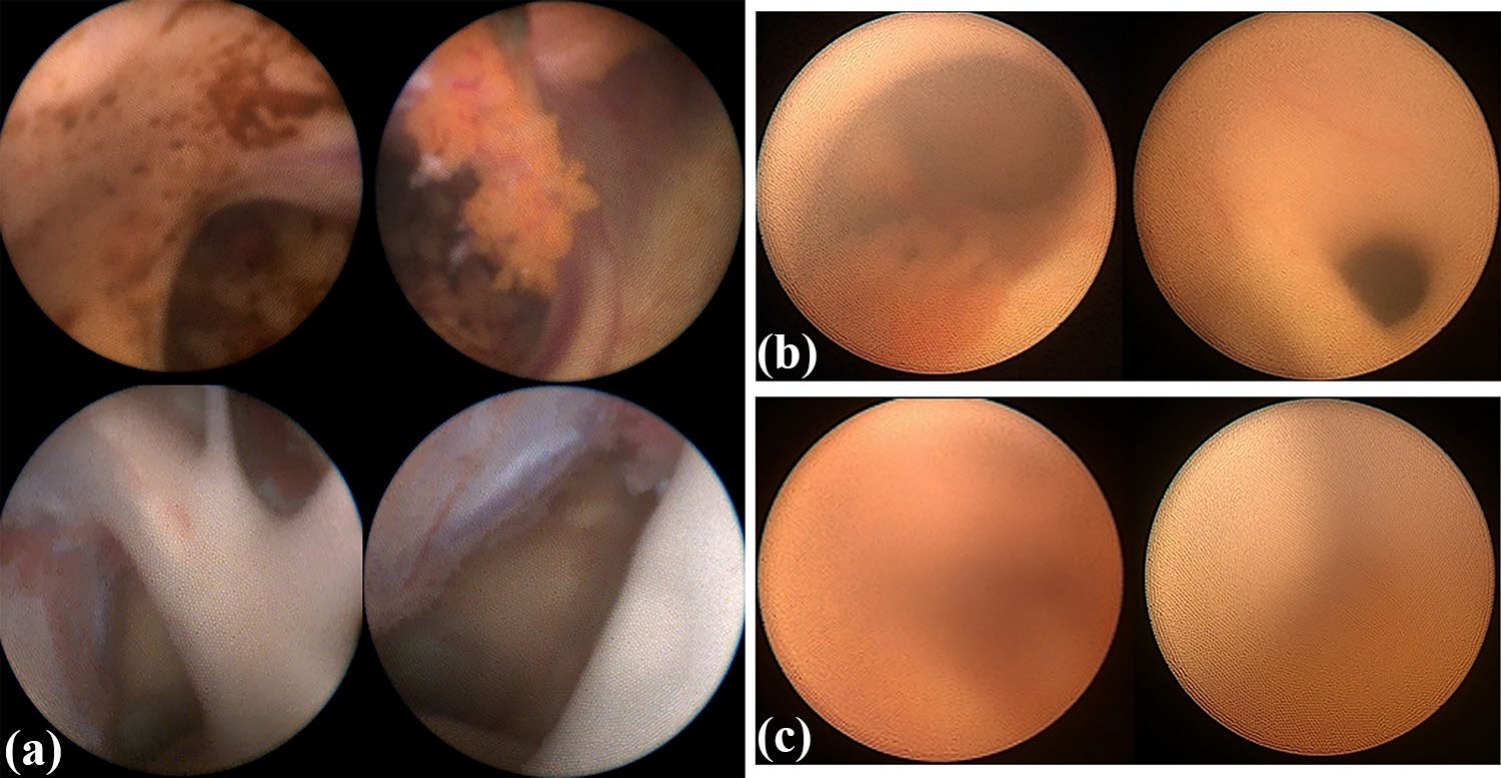
Visualization Grading of intraoperative ShuntScope achieved image quality. **a** Excellent image: The intraventricular landmarks are absolutely obvious and simply recognizable. The quality of the image is magnificent. Vascular structures such as choroid plexus, thalamostriate vein, septal vein, or basilar artery are definable. **b** Medium image: However, the suboptimal image quality is satisfactory for intraventricular orientation Foramen of Monro, choroid plexus, aqueduct entrance is adequately defined. **c** Poor image: an insufficient, blear, fuzzed image with highly restricted recognizability of intraventricular anatomy. The achieved image is not usable for intraoperative orientation during catheter placement

The intraoperative videos were analyzed retrospectively. The referring cases were grouped depending on the endoscopic visualization achieved during surgery. Stratification resulted in either one of three classifications (Fig. 2):

- *Excellent quality:* clear identification of choroid plexus, ventricular foramen, thalamostriate or septal veins, and basilar artery, if applicable. No blurring or bleeding-associated visual deterioration occurred.
- *Medium quality:* suboptimal visualization with temporary blurring or bleeding-associated visual deterioration. The choroid plexus and ventricular foramen were still identifiable.
- *Poor quality:* persistent blurry or fuzzed image with highly restricted orientation. Intraoperative orientation and identification of anatomical landmarks were impossible.

The final positioning of the ventricular catheter was evaluated using postoperative radiological studies (MRI, sonography). Therefore, the modified grading system proposed by Hayhurst et al. [6] was applied:

- *Grade I:* optimal placement of the ventricular catheter tip free-floating in CSF. No contact with the ventricular wall. No connection with the choroid plexus. In trans-cystic VC placement, there is no contact of VC with the cyst wall.
- *Grade II:* suboptimal positioning, including cross-over placement or direct contact with either the ventricular wall, cyst wall, or the choroid plexus.
- *Grade III:* misplacement of the ventricular catheter in the extra-ventricular space or occurrence of additional morphological postoperative findings, such as hemorrhage.

All data was compiled using SPSS (IBM Inc., Armonk, NY, USA). The primary outcome parameter was a statistical analysis regarding intraoperative visualization quality and postoperative Hayhurst gradings. For the secondary outcome parameter, clinical follow-up was evaluated regarding the incidence of surgical shunt revisions due to proximal catheter occlusion. The statistical level of significance was set at *p* < 0.05.

## Results

### General information on the study population

Between 01/2012 and 01/2022, 65 procedures under ShuntScope guidance were performed in 51 children. The mean age at surgery was 5.1 years (6 days–17 years). The male-to-female ratio was 2.2:1. Most frequent indication for CSF restoration surgery was secondary hydrocephalus due to tumors or cysts (51%). Procedures included Rickham reservoir placement (29.2%), shunt revisions (21.5%), trans-aqueductal stent placement (13.8%), and first-time shunting (12.3%) as the most common strategies. Detailed information on the study population and the procedures performed are shown in Tables 2, 3, 4 and 5.

**Table 2:** Descriptive statistics of parameters by the image quality

			**Procedures** ***N*** = **65/patients** ***N*** **= 51**			***P*** **value/significance**
			**Excellent image (27)**	**Medium image (28)**	**Poor image (10)**	
**Female/male**	**16/35**		8/19	13/15	4/6	**0.485**
**Age**	**Mean age of 5.1 years (6 days to 17 years)**		2.8 years	5.71 years	7.8 years	**-**
**Pathology**	**Tumor/cyst-related HC with occlusive/malresorptive component**	**26/51**	6	14	6	**0.190**
	**HC communicans**	**24/51**	11	11	2	
	**Isolated IV ventricle after shunting**	**8/51**	5	2	1	
	**Pseudotumor cerebri**	**2/51**	0	1	1	
	**Craniosynostosis related HC**	**1/51**	1	0	0	
	**Dandy-Walker malformation-related HC**	**1/51**	1	0	0	
	**Intrinsic aqueduct stenosis**	**2/51**	2	0	0	
	**Posttraumatic HC with subdural hygroma**	**1/51**	1	0	0	
**Modified Hayhurst score**	**Grade I**	**50/65**	21	23	6	**0.290**
	**Grade II**	**14/65**	6	5	3	
	**Grade III**	**1/65**	0	0	1	
**Proximal shunt failure rate**	**VC occlusion due to tumor progress**	**2/65**	2	0	0	**0.333**
	**VC misplaced**	**1/65**	0	0	1	

Analysis of correlation between image quality and accuracy of catheter position. Chi-square test of independence, a *p*-value ≤ 0.05 indicates a significance level

**Table 3:** Distribution of the ShuntScope-assisted procedures

**ShuntScope assistance for**	**Number of cases**	**Percentage (%)**
Burr hole reservoir insertion	19	29.2
Shunt revision surgery	14	21.53
Trans-aqueductal stent placement	9	13.84
First-time shunting	8	12.30
Trans-cystic VC placement	5	7.7
Trans-foraminal VC placement	4	6.15
Trans-septal VC placement	3	4.61
Placement of external VC	2	3.07

**Table 4:** Concomitantly with ShuntScope-assisted procedure, performed endoscopic procedures

**The endoscopic procedure with a standard endoscope**	**Number of cases**
Cysto-ventriculostomy	5
Endoscopic septum pellucidotomy	3
ETV	3
Endoscopic foraminotomy on foramen of Monro	2
Endoscopic inspection	2
Endoscopic choroid plexus coagulation	1
Endoscopic stomy in multiloculated HC	1

*HC* hydrocephalus, *ETV* endoscopic third ventriculostomy

**Table 5:** Summary of indications for shunt revision surgery in 51 patients

**Reason for revision surgery**	**Number of cases**	**Percentage (%)**
Postoperative infection or impaired wound healing	9	17.6
Shunt disconnection, peripheral shunt malposition	5	9.8
Ventil dysfunction, malfunction	3	5.8
Shunt malfunction due to proximal VC occlusion/dysfunction	2	3.9
Foreign body reaction	2	3.9
Extension of the abdominal catheter	1	1.9
Shunt malfunction due to proximal VC misplacement	1	1.9

### Intraoperative visualization quality

**Fig. 3:**
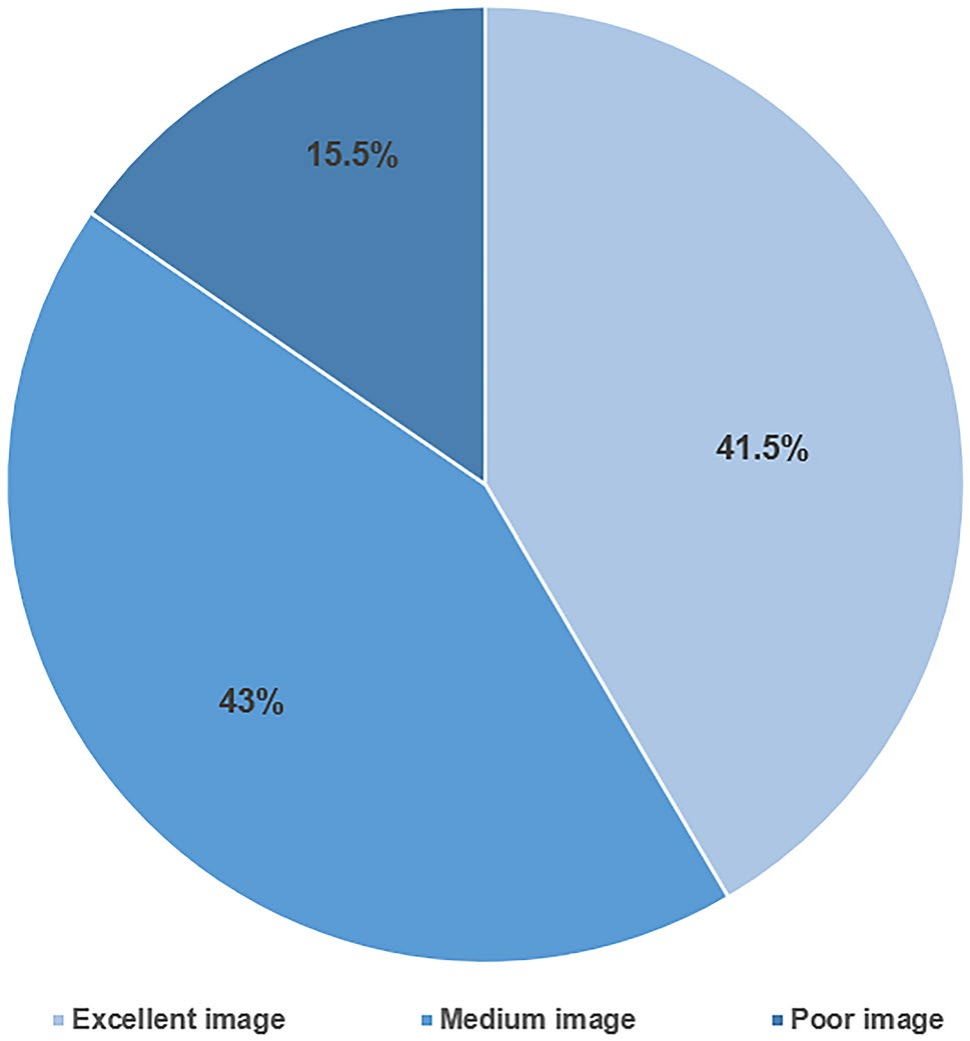
Distribution of achieved image quality during ShuntScope-assisted guidance based on visualization grading

The endoscopic visualization quality was rated excellent in 27 (41.5%) procedures. Medium quality was achieved in 28 (43%) systems. Poor visualization was seen in 10 (15.5%) operations. Exemplary results of the stratified findings are shown in Fig. 3.

### Surgical results

All but two surgeries could be completed under endoscopic guidance with the ShuntScope. In two cases of trans-aqueductal stenting (3.07%), the ventricular catheter had to be placed within the lateral ventricle due to insufficient visualization by the ShuntScope. There were no endoscopy-related intraoperative complications and no technique-related morbidity.

### Assessment of the ventricular catheter positioning

**Fig. 4:**
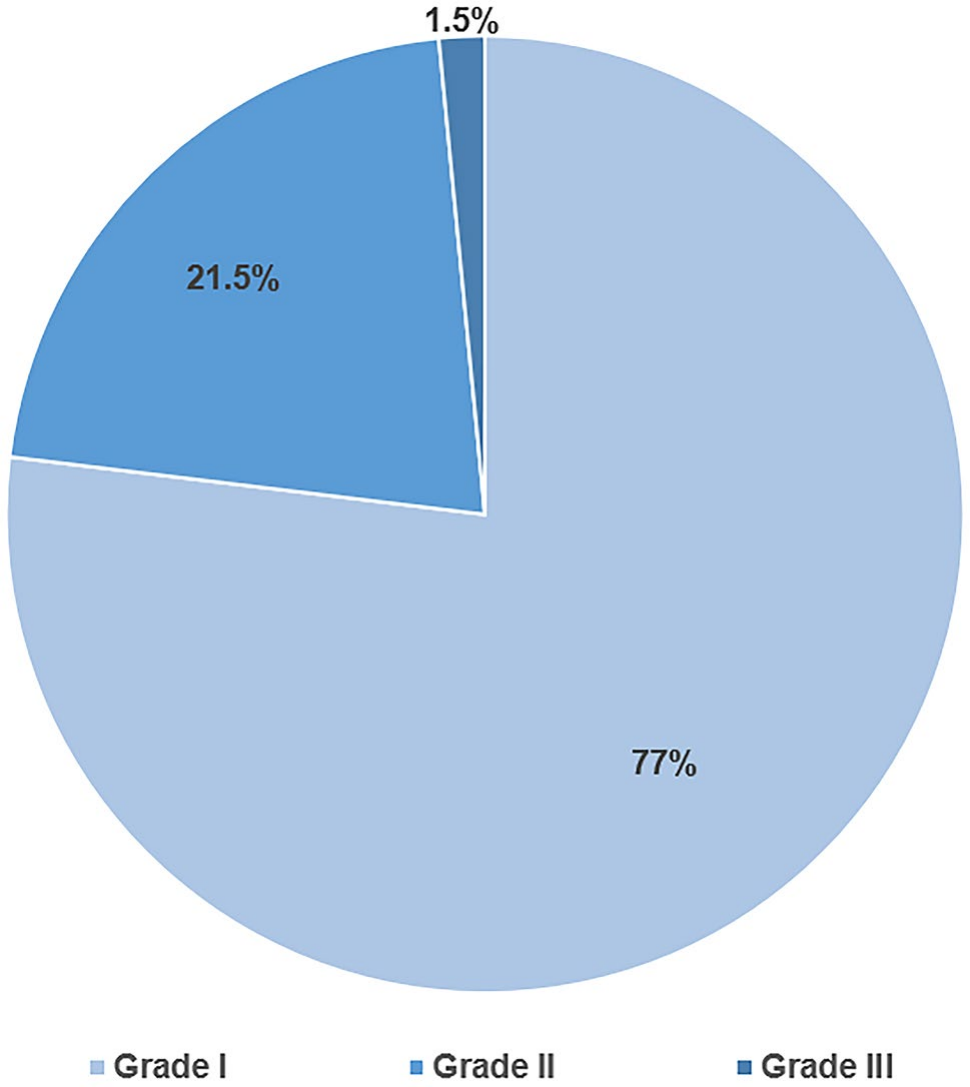
Accuracy of VC placement in the postoperative image for 65 performed procedures

**Fig. 5:**
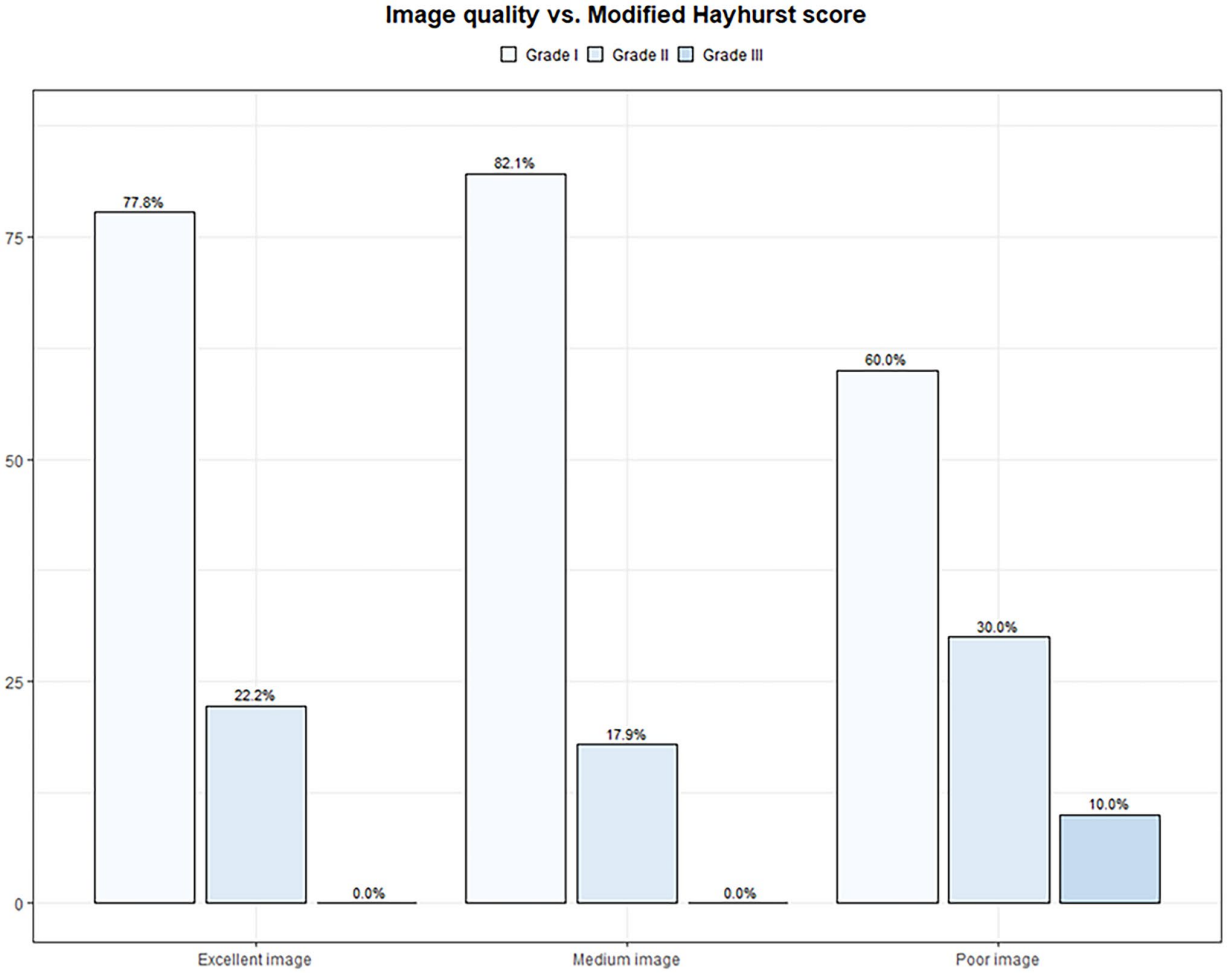
Distribution of image quality vs. Modified Hayhurst score

Evaluation of the postoperative radiological studies showed optimal ventricular catheter placement in 50 (77%) cases. Grade II placements could be found in 14 (21.5%) patients. In one (1.5%) case, postoperative MR images showed misplacement of the ventricular catheter, defined as grade III (Fig. 4). No statistical correlation between image quality distribution and positioning of the VC was found (*p*-value was 0.290) (Fig. 5).

### Distribution of image quality vs. proximal shunt failure rate

**Fig. 6:**
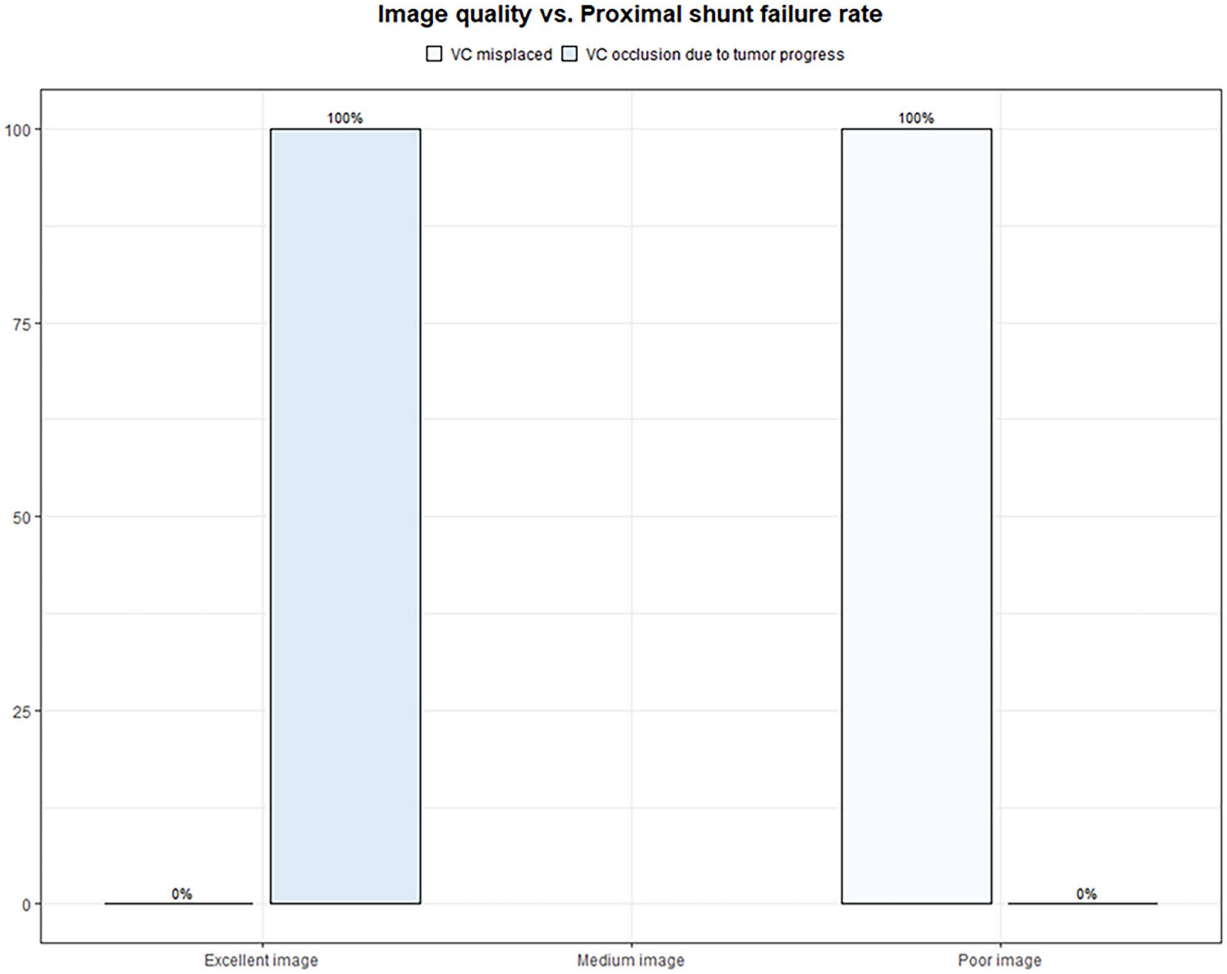
Distribution of image quality vs. Proximal shunt failure rate

Only three cases (4.61%) underwent revision surgery due to proximal catheter dysfunction. In one case, the child with a misplaced ventricular catheter grade III underwent successful surgical replacement. The intraoperative achieved image quality, in this case, was limited. Two other revision surgeries had to be conducted due to proximal catheter occlusion by tumor progress. In both cases, the intraoperative excellent image quality and correct VC placement, grade I, shown in the postoperative MR images, were obtained (Fig. 6). Consequently, in these three cases, VC had to be revised.

### Follow-up and catheter revisions

The mean clinical follow-up was 39.7 months (from 1 month to 9.5 years). Within this time frame, 23 revision surgeries have been performed in 18 of the included patients. The most frequent indications for revision surgery were postoperative wound healing disorders or infections. We did not observe a CSF leakage through the point of the punctured membrane of the reservoir during burr hole reservoir placement. The revision rate due to proximal VC dysfunction was 4.61%.

### Statistical evaluation

Statistical analyses of the correlation between image quality and the accuracy of catheter placement have been performed. The chi-squared test was used to compare the distribution of a categorical variable in the discussed group. The significance level was defined as *p* < 0.05. A summary of the results stratified after intraoperative image quality stratification is given in Table 2 and Figs. 5 and 6.

## Case illustration

**Fig. 7:**
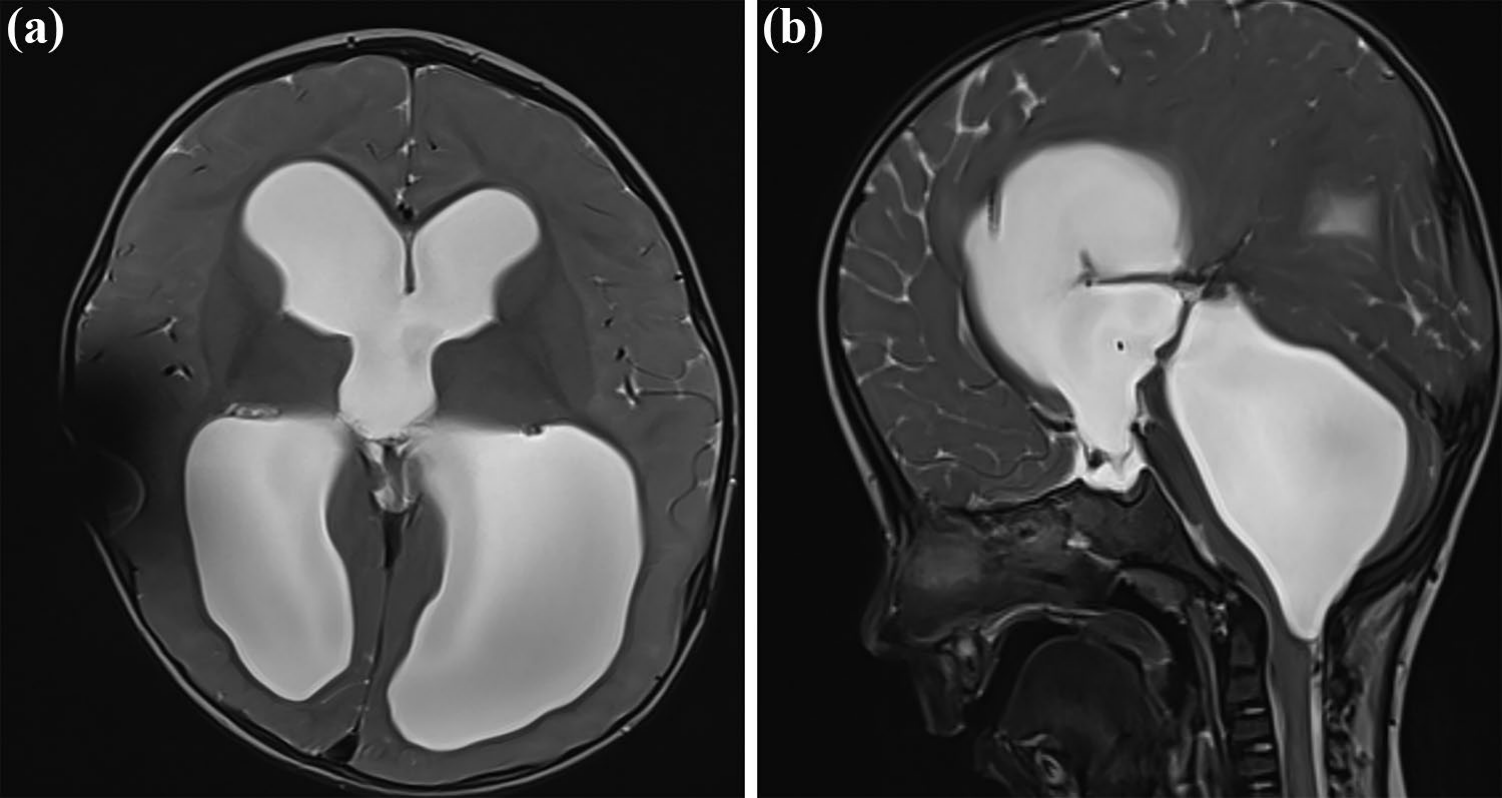
Preoperative MR images. Axial T2—(**a**) and sagittal T2-(**b**) weighted MR images. Chronic hydrocephalus with an aspect of isolating fourth ventricle after shunting

**Fig. 8:**
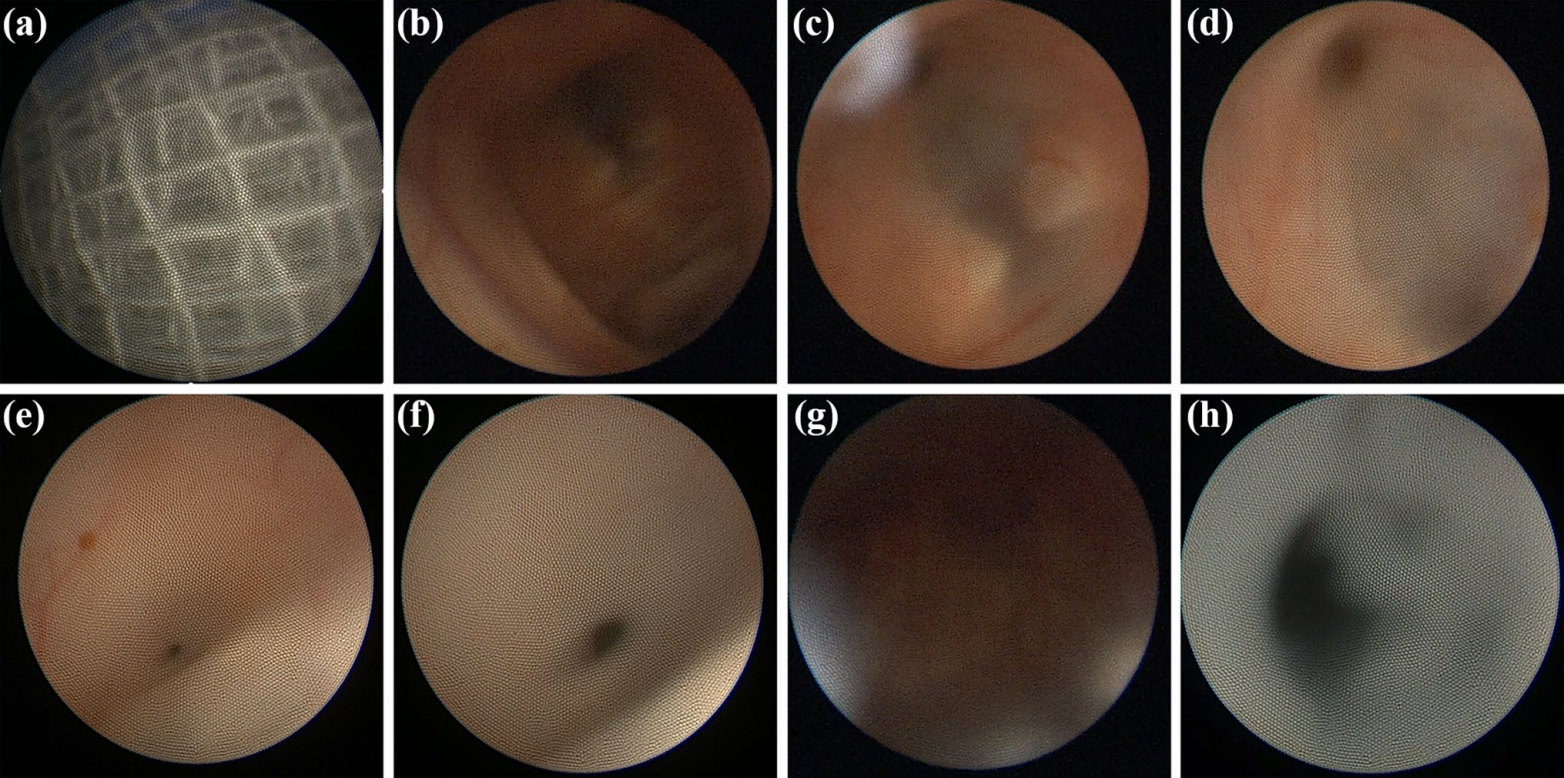
Intraoperative ShuntScope-guided images. Sequential intraoperative photographs show the ShuntScope-controlled exploration and trans-aqueductal stent placement. Sufficient image obtained with recognition of the ventral and dorsal aspect of the third ventricle; right foramen of Monro, the floor of the third ventricle with the mamillary body (**b**, **c**), infundibular recess (**d**). Endoscopic view of pathologic cerebral aqueduct; narrow, obliterated aqueductal entrance, posterior commissure (**e**, **f**). Exploration of the fourth ventricle (**g**). The ShuntScope is stepwise withdrawn, transluminal image (**h**) by correctly positioning the trans-aqueductal stent

**Fig. 9:**
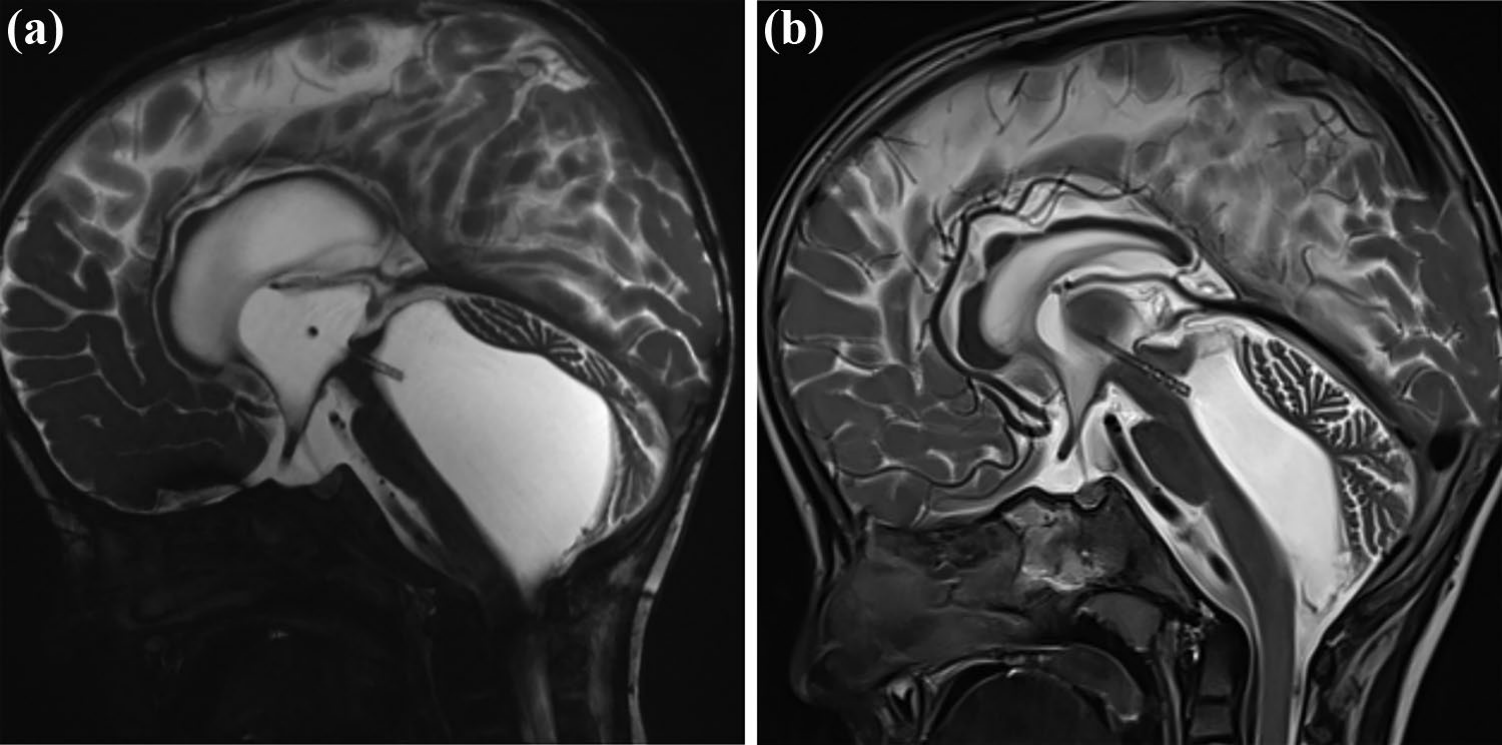
Postoperative control MR images. MR images were taken four days after surgery (**a**) and 14 months after surgery (**b**), confirming the trans-aqueductal position and functionality of the stent. The size of the fourth ventricle regressed revenant resulting in the resolving of previous brain compression. The prepontine cistern was again definable. The cerebellum was unfolded. All figures were created using Adobe Photoshop

A 7-month-old boy, initially a premature newborn, presented with post-hemorrhagic hydrocephalus. At the age of 2 months, the patient developed malresorptive hydrocephalus. VP shunting procedure was performed. The child underwent a few shunt revision surgeries in the further course due to shunt dysfunction. After 13 months of shunting therapy, progressive dilatation of the fourth ventricle with compression of the brainstem ventrally and cerebellum dorsally developed. Lack of flow-void sigh through the aqueduct and outflow disturbance correlated with the aspect of isolated fourth ventricle after shunting (Fig. 7). The decision of ShuntScope-assisted trans-aqueductal stent placement was made (Fig. 8), Video 1. Postoperative MR images were obtained four days after surgery. The length of the proximal catheter running through a very narrow aqueduct and placed in the upper part of the fourth ventricle was short and not deeply inserted, as initially intended. However, because of clinical and radiologic improvements seen in control MR images, no indication for revision was made. The MR images obtained at 14-month follow-up showed the functionality of the stent: the size of the fourth ventricle regressed. The prepontine cistern was again definable. The cerebellum was sufficiently unfolded (Fig. 9). The patient was doing fine.

## Discussion

Shunting for cerebrospinal fluid (CSF) restoration in treating hydrocephalus in pediatric patients is a standard procedure. Ventricular catheter tip location and ventricular catheter environment are crucial for clinical improvement and shunt survival [18]. The accurate position of the VC plays a significant role and contributes remarkably to the long-term patency of the shunt, even if statistical significance is not always achievable.

Regardless of advances in improving valve design and prevention of infection, the failure rate by the placement of a ventricular catheter during the shunting procedure has not been changed. The shunt failure in the first year after placement reported in the literature varies from 20 to 50% [14, 19–22]. Catheter obstruction due to in-grow of the choroid plexus and occlusion of the proximal catheter lumen is estimated as one of the causes leading to shunting dysfunction [1, 7, 17, 22, 23]. Some studies have investigated that accurate ventricle catheter position correlates with and influences the shunt survivor rate [24]. The standard freehand technique for shunting procedures based on anatomical landmarks is generally considered feasible and straightforward. Daily routine shows that consistent efforts are taken; an incorrect or suboptimal VC position is not a rare condition and varies significantly in reported series, reaching up to 45% [1–3]. Albright et al. [25] achieved an excellent VC tip position only in 55% of 114 studied pediatric patients. Technical advances in intraoperative image guidance for improving the accuracy of VC placement have been introduced in the last years. Neuronavigation, neuroendoscopy ultrasound, electromagnetic-based stereotaxy, or smartphone-assisted guiding tools have been implemented and reported as promising methods for increasing the accuracy of VC placement [4–9]. However, these randomized studies have shown no significant benefit using the abovementioned supportive techniques.

The high accuracy rate of ventricular catheter placement, which reached 93.2%, was achieved by Pang and Grabb [26]. They pointed out that proper positioning for a coronal shunt, in turn, depends on the ventricular catheter length and target coordinates. Using freehand passage guided by simple stereotactic coordinates based on visible and palpable surface anatomy, the catheter length was calculated based on bone landmarks on skull radiographs. Of 160 children undergoing ventriculoperitoneal shunt insertion using this technique, only three required catheter revision during a mean follow-up period of 39 months. Kestle et al. [7] presented a multicentre randomized trial. The endoscopy was applied to 194 hydrocephalus patients. The authors concluded that endoscopic insertion of the initial ventriculoperitoneal shunt in pediatric hydrocephalus did not reduce the incidence of shunt failure at 1 year, estimated at 42% in the endoscopic insertion group and 34% in the non-endoscopic group at 1 year. Roth and Constantini [16] described their experiences with 16 children by whom the endoscope-assisted shunting was performed. They used NeuroPEN Neuroendoscope (Medtronic PS Medical, CA, USA). Fourteen procedures were technically successful. The catheter was located adequately on postoperative imaging in 13 procedures. With the constant development of neuroendoscopic technology, the intra-catheter endoscope, the so-called ShuntScope, has increased attention over the last years. The versatile application possibilities of the intra-catheter endoscope and the advantages of this technique have been reported previously [10–13]. The modified burr hole reservoir was performed on 12 patients [12]. The optimal positioning of the catheter was achieved in 11 of 12 cases. The authors postulated that using the intra-catheter endoscope combined with the modified burr hole reservoir provided a sufficient technique for accurate and safe VC placement. In the study by Senger et al. [13], the ShuntScope-guided precise catheter placement was achieved in 26 of 27 studied patients. The postoperative imaging studies demonstrated catheter tip placements analogous to the intraoperative finding. In another prospective study by Issa et al. [15], the ShuntScope technique was used in 29 patients. Compared to the freehand method, the success rate for optimal VC placement was significantly higher (93.1% vs. 67%), combined with lover revision and complication rate.

The current presentation illustrates our experiences with ShuntScope-assisted shunting caused by various CSF pathway impairments in children we gained over the last 10 years in our institution. Firstly, we would like to stress the value and the benefit of ShuntScope guidance in shunting procedures. Contrary to other adjuncts mentioned earlier, ShuntScope represents an intraoperative, real-time diagnostic option. The real image verification and a possibility of an intraventricular field exploration with simultaneous intraoperative assessment and correction of the placed VC are indeed beneficial. High-image resolution with 10,000 pixels enables receiving a reasonable image. Excellent and medium image quality, sufficient for intraventricular orientation with visibility of the prominent intraventricular landmarks, such as the Foramen of Monro and choroid plexus, was achieved in 55 procedures (84.5%). In 10 procedures (15.5%), the image quality was limited and unreliable for intraoperative guidance. ShuntScope failure in our series was assessed at 4.61%. In two cases, the ventricle catheter had to be revised due to tumor progress related to VC obstruction. In the third case, the VC was placed initially under limited ShuntScope visualization. The postoperative image showed the extra-ventricular misplacement of the VC in this case. The child underwent revision surgery. There was no technique-related morbidity.

Under some circumstances, such as post-infectious, post-hemorrhagic, or leptomeningeal carcinomatosis related to CSF impairment, CSF contains a much higher level of proteins, which can lead to cloudy, limited image. ShuntScope does not allow for concomitant irrigation, contrary to traditional neuroendoscopes; therefore, the image obtained with the ShuntScope system may be limited and unreliable under adverse conditions.

Interestingly, under these poor conditions, in our series, the catheter was correctly placed in 9 procedures (6 with grade I and 3 with grade II), verified radiologically. In one case mentioned above, the ventricular catheter was placed extra-ventricular under a limited image. On the other hand, even if sufficient ShuntScope-assisted image is achieved, an intended placement may be difficult. In our series, in one case, during trans-aqueductal stenting procedures, the intended therapy had to be abandoned and switched to VC placement into the lateral ventricle because of uncontrollable anatomical circumstances. In another case, because of the limited ShuntScope image, the standard Oi HandyPro pediatric endoscope was used for AS placement. We found a correct VC position in the MR images. Based on achieved radiologic data, the optimal VC placement was verified in 50 of 65 procedures (77%), where the tip of the VC was placed without touching the wall of the ventricle or cyst. (grade I). The suboptimal intraventricular position was seen in 14 cases (21.5%) (grade II). The extra-ventricular VC placement (grade III) was seen in one case. No statistical correlation between ShuntScope-guided image grade and the accuracy of the catheter placement was observed (*p*-value was 0.290). Looking through the literature, suboptimal VC placement is considered a significant risk factor for proximally related shunt dysfunction and failure [6, 27, 28]. CSF impairment in children varies significantly from that of adults. Of course, in-depth knowledge of intraventricular anatomy is of paramount importance for the safety of shunting procedures. Profound anatomical orientation plays an even more significant role when we are faced with distorted anatomies, such as tumor-related obstruction, ventricle asymmetry due to compression or exophytic tumor growth pattern, slit ventricle syndrome, or other anatomical aberration, such as septal perforation or thinning of fornix observed in chronic or multiloculated hydrocephalus. All these conditions may lead to incorrect VC placement. Particularly in the circumstances mentioned above, ShuntScope assistance, in our opinion, should be considered. In summary, ShuntScope-assisted technique remarkably helped good VC positioning in selected cases. However, the accuracy of VC placement has not guaranteed clinical improvement solely in various etiology treatments, as presented. In our series, we applied the ShuntScope technique in most of the studied cases and added important information regarding intraventricular orientation and the final positioning of the ventricular catheter. No technique-related morbidity was observed. Based on our results, we highlight discussed aspects of the ShuntScope-assisted technique and encourage the integration of this technique into others’ practice.

## Conclusions

ShuntScope represents a valuable intraoperative, real-time diagnostic option in shunting procedures in children. It has radiologically good results with an accurate VC placement rate, estimated in our series at 77%. Particularly in complex cases and challenging circumstances, suboptimal positioning can be avoided. ShuntScope efficacy was evaluated at 84.6%. The insertion of ShuntScope is recommended as a supportive adjunct in treating hydrocephalus in pediatric patients, and its application should be considered more frequently.

## Supplementary Information

Below is the link to the electronic supplementary material.

**Supplementary file1** Video documentation of ShuntScope-guided transaqueductal stent placement (MP4 75963 KB)

## Data Availability

Raw data are not provided. All data are summarized in the provided graphics and tables. The raw datasets used and/or analyzed during the current study are available from the corresponding author upon reasonable request.

## Abbreviations

CSF: Cerebrospinal fluid
MR images: Magnetic resonance images
VC: Ventricular catheter
VP: Ventriculoperitoneal
AS: Aqueductal stent
HC: Hydrocephalus
ETV: Endoscopic third ventriculostomy
